# Effects of Intravenous Versus Intraosseous Adrenalin Administration on Morbidity and Mortality After Out-of-Hospital Cardiac Arrest: A Systematic Review

**DOI:** 10.3390/medicina61040680

**Published:** 2025-04-07

**Authors:** Sjaak Pouwels, Emschka Johannes, Juan Pablo Scarano-Pereira

**Affiliations:** 1 Department of Surgery, Campus Detmold, Klinikum Lippe, Bielefeld University, 32756 Detmold, Germany; 2 Department of Intensive Care Medicine, Elisabeth-Tweesteden Hospital, 5022 GC Tilburg, The Netherlands; 3 Department of Surgery, Marien Hospital Herne, University Hospital of Ruhr University Bochum, 44653 Herne, Germany; 4 Department of Emergency Medicine, University Hospital Brussels, 1090 Brussels, Belgium; emschkaj@gmail.com; 5 Faculty of Medicine, Universidad Complutense de Madrid, 28040 Madrid, Spain; juanpablosca@hotmail.com

**Keywords:** intraosseous access, intravenous access, critical care, emergency medicine, OHCA, out-of-hospital cardiac arrest

## Abstract

*Background and Objectives*: Out-of-hospital cardiac arrest (OHCA) is a common manifestation of heart disease and a leading cause of death in western societies with an overall survival rate of 10%. Guidelines generally prefer the peripheral intravenous (IV) access as the first option for OHCA patients, leaving the intraosseous (IO) route for patients in which IV access is not feasible or unsuccessful. This systematic review will purely focus on the clinical differences between adrenaline administered via the IO route compared to the IV route and its effects on morbidity and mortality after OHCA. *Materials and Methods*: A multi-database (PubMed, Medline, Embase, and The Cochrane Library) was performed and was searched between the earliest date of each database and 16 February 2024. For data extraction, a structured checklist was used, including type of study, the number of patients, age, gender, Return of Spontaneous Circulation (ROSC), associated morbidity, mortality, neurological, and general outcome. *Results*: The initial literature search produced 1772 results. After screening for title and abstract, a total of nine studies were included in our systematic review. Of these studies, six were retrospective cohort studies, one prospective study, and two sub-analyses of previous randomized trials. Due to significant heterogeneity, a meta-analysis was not performed. *Conclusions*: In our systematic review we have found a small number of studies comparing IV and IO adrenaline administration during cardiac arrest. Due to significant heterogeneity, a meta-analysis was not performed and no firm conclusions could be drawn about which route of adrenalin administration leads to better outcomes.

## 1. Introduction

Out-of-hospital cardiac arrest (OHCA) is a common manifestation of heart disease and a leading cause of death in Western societies, with an overall survival rate of 10% [1,2]. In Europe alone, it is estimated that 67 to 170 people per 100,000 inhabitants suffer from OHCA annually [3]. The high prevalence and the poor outcomes of OHCA highlight a critical need to further improve the management of these patients [2].

According to the latest European and American guidelines for cardiopulmonary resuscitation, the first steps to manage OHCA are to perform cardiopulmonary resuscitation (CPR) followed by a rhythm assessment to determine whether the cardiac arrest is shockable or not [3,4]. If the rhythm is not shockable or if the patient fails to defibrillate, intravenous access is recommended for the early administration of emergency pharmacotherapy [4].

Guidelines generally prefer peripheral intravenous (IV) access as the first option for OHCA patients, leaving the intraosseous (IO) route for patients in whom IV access is not feasible or unsuccessful [3,4]. Although the IV route has traditionally been the preferred method for providing resuscitation medication, the IO route has recently grown in popularity in virtue of its advantages [1,4]. IO access has not only been shown to be a quicker route to successfully cannulate a vein in cardiovascular collapse but also to reduce the number of unsuccessful attempts [2,5,6,7,8]. Albeit some authors disagree with the former idea, this relative ease of IO access has encouraged different medical services to turn this procedure into their first choice of access in the event of an OHCA [4,8,9].

Pharmacokinetic models on animals have shown similar peak drug concentrations (Cmax) and time-to-peak drug concentrations (Tmax) between drugs administered via sternal IO or humeral IO and a central vein catheter [10,11]. Burgert et al. [11] investigated drug concentrations and their pharmacological profiles in a swine model after a traumatic reanimation model. Cmax after, respectively, sternal IO, Humeral IO, Tibial IO, and IV was 626 ± 152 ng/mL, 474 ± 76 ng/mL, 426 ± 63 ng/mL, and 870 ± 165 ng/mL. Tmax was reached after, respectively, 141 ± 17 s, 150 ± 18.5 s, 223 ± 17 s, and 154 ± 17.8 s [11].

Although tibial IO seems to be less effective, there are other studies that support its use [12]. Return of spontaneous circulation (ROSC) has also been achieved in different animal models with no statistically significant difference in timing between tibial IO and IV access [11,13].

Even though several clinical studies have also demonstrated that IO and IV access are comparably effective in OHCA patients [7,8,9,14], recent studies have raised questions about the efficacy of IO access on OHCA patients based on lower survival rates at all stages of patient care [5,6], poorer neurological outcome (6), and worse ROSC rates [1,2,6]. Thus, it is essential to further assess the efficacy of IO access relative to IV access in cardio-pulmonary resuscitation. Granfeldt et al. [9] recently performed a systematic review on drug administration via IO and IV route in OHCA patients in which pooled results from four observational studies favored IV access with very low certainty of evidence and the subgroup analyses of two randomized clinical trials found no statistically significant interaction between the route of access and study drug on outcomes. This systematic review will purely focus on the clinical differences between adrenaline administered via the IO route compared to the IV route and its effects on morbidity and mortality after OHCA.

## 2. Materials and Methods

A systematic literature search was conducted using the PICO(T) acronym (patient, intervention, comparison, outcome, and time). The patient population of interest was all adult patients with an OHCA. The intervention studied was intravenous administration of adrenaline compared with intraosseous administration of adrenaline. Outcome measures of interest were morbidity, mortality (in particular prehospital and in-hospital mortality), return of spontaneous circulation (ROSC), and neurological outcome [9,14].

A multi-database (PubMed, Medline, Embase, and The Cochrane Library) was performed using the following search string: ((Intravenous OR Intraosseous) AND (adrenaline OR epinephrine)) AND (cardiac arrest OR cardio-pulmonary resuscitation OR out-of-hospital cardiac arrest OR OHCA). Each database was searched between the earliest date of each database and 16 February 2024.

Authors EJ and JPSP individually screened and selected studies on the basis of title and abstract. After primary selection, each author reviewed the selected studies’ full text to determine suitability for inclusion based on the established selection criteria according to the Preferred Reporting Items for Systematic Reviews (PRISMA) guidelines [15]. For further eligible studies, cross-references were screened. All disagreements were discussed with each other or with the senior author (SP) until consensus was reached.

All original articles (in particular randomized controlled trials (RCT), prospective and retrospective cohort studies, and before–after studies) published in the English language were included. Review papers and non-English language articles were excluded. Due to the inconsistent reporting of outcome measures and the small number of included studies (e.g., patients), a meta-analysis was not performed. For data extraction, a structured checklist was used, including the type of study, the number of patients, age, gender, ROSC, associated morbidity, mortality, neurological and general outcomes.

Methodological quality of the included studies was rated using the Newcastle–Ottawa Scale (NOS) for non-randomized trials [16]. The NOS uses a stars system for a quick visual assessment of the methodological quality of studies. A maximum of nine points can be assigned for the least risk of bias in the following three domains: (1) selection of study groups (4 points), (2) comparability of groups (2 points), and (3) ascertainment of exposure and outcomes (3 points). Two authors (EJ and JPSP) separately assessed the NOS of the included studies. A Cohen’s kappa score was calculated to determine the level of agreement between the authors [17].

## 3. Results

**Figure 1:**
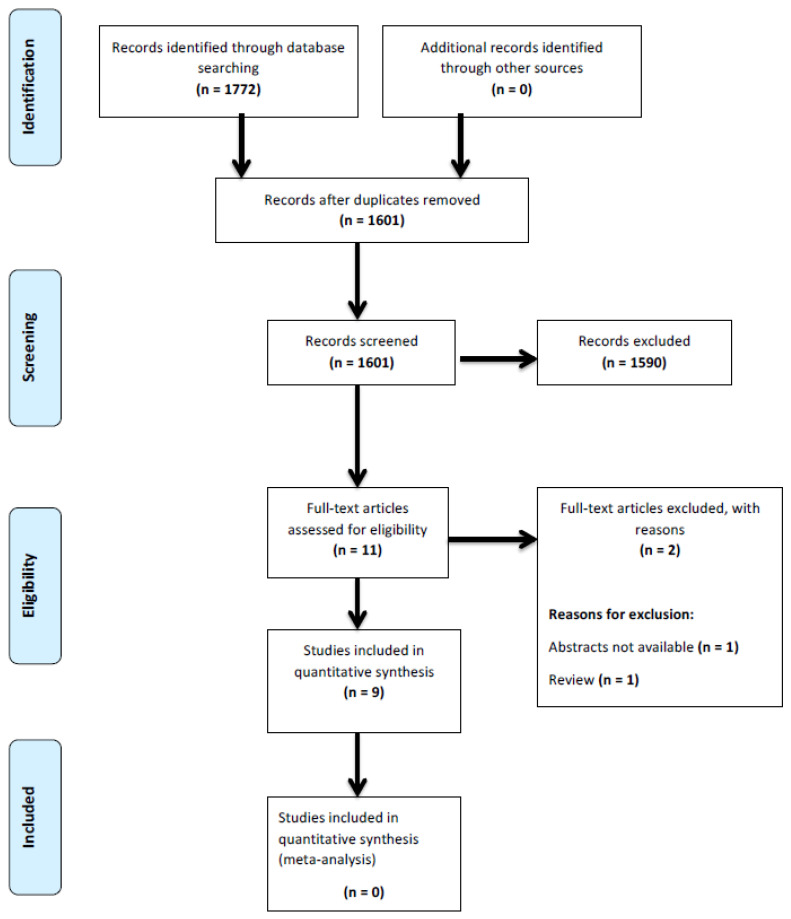
PRISMA flowchart of the included studies.

The initial literature search produced 1772 results, including 171 duplicates. After screening for title and abstract, 11 potentially relevant studies were found and underwent a full-text critical appraisal. Of these studies, two had to be excluded from the analysis since one was a review article and the other did not have a full-text copy available. In total, 9 studies were included in the systematic review. Figure 1 shows the PRISMA flowchart of the search strategy, and Table 1 gives an overview of the characteristics of the included studies. The methodological quality of the included studies is shown in Table 2 and ranged between moderate and good quality. A Cohen’s Kappa of 0.68 indicated a good agreement between authors EJ and JPSP.

### 3.1. Comparison Between IO and IV Access in OHCA

Feinstein et al. [1], in a retrospective cohort study hypothesized that IO access may be a less effective route than IV for drug administration during resuscitation of adults with OHCA. After excluding 364 patients due to age less than 18 years, missing information or a Physician’s Orders for Life-Sustaining Treatment (POLST) status, 1800 cases of OHCA were analyzed [1]. Of the group they studied, 1525 had an IV access and 275 had an IO access. In the unadjusted analyses performed, the patients in the IO group compared to the IV group were less likely to survive to hospital discharge (14.9% vs. 22.8%, respectively, *p* = 0.003), achieve sustained ROSC (43.6% vs. 55.5%, *p* < 0.001) or survive to hospital admission (38.5% vs. 50.0%, *p* < 0.001). After the multi-variable adjusted analyses there was no association of IO access with survival to hospital discharge (CI 95%, OR 0.81, 0.55, 1.21, *p* = 0.31). On the contrary, there was a lower likelihood of ROSC (OR = 0.67, 0.50, 0.88, *p* = 0.004) and survival to hospital admission (OR = 0.68, 0.51, 0.91, *p* = 0.009) in the IO group [1].

The study conducted by Mody et al. [2] also looked at a possible correlation between attempts to intravenous vs. intraosseous access and survival to hospital discharge. Of 19,731 patients, 15.5% (n = 3068) had an IO access attempt, of which 2975 were successful, vs. 84.4% (n = 16,663) of patients in whom an IV access was attempted and of which 15,485 were successful [2]. They found the unadjusted rate of survival to hospital discharge to be lower in patients receiving an IO access compared with those receiving an IV access (4.6% vs. 5.7%, *p* = 0.01). After adjusting for different factors (age, sex, initial cardiac arrest rhythm, bystander CPR, public location, witnessed status and emergency medical services (EMS) response time interval), there was no difference in survival to hospital discharge between attempted IO access vs. attempted IV access (OR 0.88, 95% CI 0.72–1.09, *p* = 0.24) [2]. When they performed a propensity score matched cohort between subjects with attempted IO and those with attempted IV access, there was a significantly lower rate of survival to hospital discharge (4.6% vs. 5.9%) in the attempted IO group [2]. Additional adjustments for initial access interval, initial drug administration interval, or in-hospital procedures did not meaningfully change the model results. After analyzing initial successful access only, there was no difference in survival to hospital discharge between IO and IV access. But when comparing outcomes of eventual successful access, the IO group had significantly lower survival to hospital discharge (unadjusted rate: 4.2% vs. 5.9%; adjusted OR 0.77, 95% CI 0.63–0.93, *p* = 0.005) [2].

According to Zhang et al. [5] IV administration of adrenaline for treatment of OHCA of presumed cardiac etiology would result in an increased likelihood of survival to hospital discharge, among other endpoints, compared to the IO route. This group analyzed the differences between the IV route and the IO route for adrenaline administration survival to hospital discharge being their primary outcome. OHCA patients had increased survival to hospital discharge if they received IV adrenaline compared to those receiving IO adrenaline (5.8% vs. 3.1%, *p* < 0.05). Same results were seen for the secondary outcomes, which were as follows: prehospital ROSC (24.5% vs. 17.8%, *p* < 0.05) and favorable neurological outcome at discharge (4.3% vs. 1.8%, *p* < 0.05) [5]. After adjusting for known confounders, the OR of the IV route for survival was 1.468, 95% CI, 1.264–1.705, taking the IO route of adrenalin administration as a reference. Similarly, in the propensity score matched cohort analysis, the OR for survival to hospital discharge was 1.430, 95% CI, 1.164–1.757, leading to the conclusion that adrenalin administration via the IV route was associated with better outcomes in OHCA patients compared to the IO route [5].

Baert et al. [14] looked at survival to hospital discharge or at 30 days, and some of the secondary endpoints included ROSC and neurological outcome measured at hospital discharge or day 30. After unadjusted analyses in the IO group there were less ROSC (19.7% vs. 27.7% *p* < 0.001) and less survival at day 30 (1.9% vs. 3.8%, *p* < 0.001) and no difference regarding good neurological outcome in the two groups (81.8% vs. 72.7%, *p* = 0.343). After calculating propensity scores there were still lower rates of ROSC (19.8% vs. 25.3%, *p* < 0.001) in the IO group, but there was no significant difference in the survival rate at discharge or day 30 (1.8% vs. 2.4% *p* = 0.266) nor in neurological outcome (85.2% vs. 65.7%, *p* = 0.082) between both groups [14].

### 3.2. Return of Spontaneous Circulation (ROSC) as Primary Outcome Measurement

Clemency et al. [7] considered as a primary outcome ROSC by time of arrival at the emergency department using two primary variables, namely the first access type attempted and the route of administration for the first dose of parenteral epinephrine. The analysis using the first attempted parenteral access showed the success rate for IO access to be similar to IV access, with rates of ROSC at emergency department arrival of 19.9% vs. 19.7%, *p* = 0.01 [7]. Noticeable is the significant superiority of the IO first attempt success rate, 94.8% vs. the IV first attempt success rate of 81.6% (*p* < 0.01). When looking at the first epinephrine dose administered as a variable, rates of ROSC at time of arrival at the emergency department were 20.9% when epinephrine was administered via the IV route first vs. 18.6% when administered via the IO route first (OR 0.86; 95% CI: 0.66–1.13) [7]. The study performed by Nguyen et al. [18] also considered ROSC was considered the primary outcome and showed that there was a significant difference in favor of the IV access group when achieving ROSC compared to the IO group (45.1% vs. 25.7%). When an intention-to-treat analysis was performed, 42.5% of patients had ROSC in the group where IV access was attempted first vs. 26.6% when IO was attempted first (*p* < 0.001) [18].

### 3.3. Neurological Outcome at Hospital Discharge

In Kawano et al. [6], the primary outcome was a favorable neurological outcome at hospital discharge in the IO access vs. the IV access group. Among those with IO access, 1.5% had a favorable neurological outcome vs. 7.6% in the IV access group. The Hosmer–Lemeshow goodness-of-fit test conducted to assess the overall performance for favorable neurological outcome was not significant [6]. The calculated area under the receiver operating characteristic (ROC) curve to test the discrimination for the same outcome in this model was 0.86 (95% CI 0.85–0.88). The multivariable regression models showed that IO access was associated with a decreased probability of favorable neurological outcome compared to the IV access (OR 0.24; 95% CI 0.13–0.46). The same results were seen after conducting a propensity score matching in which IO access was associated with a decreased probability of favorable neurological outcome (OR 0.23, 95% CI 0.10–0.52). Similar negative associations between IO vascular access and neurological outcome could be found after analyzing across 50 multiply imputed data sets (OR 0.22, 95% CI 0.11–0.41). All the analyses demonstrated a negative correlation between IO vascular access and all three outcomes: ROSC, survival at hospital discharge, and favorable neurological outcome at discharge [6].

### 3.4. Comparing IO and IV Access as Sub-Analyses from Other Trials

In a prospective parallel study design, Tan et al. [19] had ROSC as a primary outcome and considered outcome survival to 30 days post-arrest/discharged alive and survival with good neurological outcome as secondary outcomes. The study design used an “IV only” group and an IV + IO group. In the first group up to two attempts were made for an IV access at the scene, and in case it was not successful, the crew was required to scoop and run. In the second group, if IV access was unsuccessful after two attempts, the crew could perform up to two IO vascular access attempts, and if it was not successful, the crew was eventually required to scoop and run [19]. The IV + IO arm compared to the IV only arm presented no difference in obtaining ROSC (OR 0.99, 95% CI: 0.75–1.29). Similarly, there was no difference in survival > 30 days post-arrest (IV only 8.4% vs. IV + IO 4.9%, *p* = 0.027) or survivability with good neurological outcome (IV only 3.4% vs. IV + IO 4.0%, *p* = 0.630). Other findings in this study show a significant correlation in adding IO to IV leading to better vascular access and faster adrenaline administration; however, without significantly improving ROSC, survival to discharge, or neurological outcome [19].

In a subgroup analysis of a randomized controlled trial, Nolan et al. [8] looked at a possible correlation between adrenaline vs. placebo administered IV or IO and survival at 30 days as primary outcome, and with ROSC at hospital handover, survival to hospital discharge, and favorable neurological outcome as a secondary outcome. There was no difference in adrenaline vs. placebo given via the IO or IV route on ROSC, nor on 30-day survival or neurological outcome. The OR (adrenaline vs. placebo) for ROSC at hospital arrival in the IV group and IO group were similar (aOR 4.07; 95% CI 3.42–4.85 vs. aOR 3.98; 95% CI 2.86–5.53) with P-value for interaction 0.90 [8]. The confidence interval for survival at discharge and 30 days and favorable neurological outcome also overlapped for both the IO and IV groups. Cumulative survival to 30-day curves were higher for the IV arm than for the IO arm in both adrenaline and placebo, but with overlapping confidence intervals and no statistical evidence for interaction (*p* = 0.70 within 1 day and *p* = 0.50 over 1 day). The aHR > 1 day survival was 1.30 (95% CI 0.98–1.72) in adrenaline and 1.08 (95% CI 0.68, 1.71) in placebo. Seeing that there was no difference detected in the treatment effect of adrenaline vs. placebo on ROSC, 30-day survival, or favorable neurological outcome at discharge, it suggests the absence of any significant difference between the IO vs. the IV route of administration [8].

## 4. Discussion

This is a systematic review comparing the efficacy of IV and IO administration of adrenaline during an OHCA. In our systematic review, only a limited number of studies were identified addressing comparisons with IV and IO access in OHCA. These articles do not show a clear difference between IO and IV access on several outcome parameters. However, we do have to keep a few aspects in mind.

### 4.1. Pharmacokinetics

There is no clear benefit of IV or IO access among the included studies in this systematic review [1,2,5,6,7,8,14,18,19]. The results can be explained by a difference in pharmacokinetics when adrenaline has been administered IO compared to IV. The first attempt success rate has repeatedly been proven to be higher in IO access than in IV access. Another important factor to consider is that in IO access, adrenaline may have to traverse the bone marrow before reaching systemic circulation [20,21]. This might lead to drug sedimentation and potentially lower concentrations of adrenaline in the peripheral circulation [5,20,21]. Indeed, Von Hoff et al. [22] found that distribution volume in the central compartment was significantly lower in IO infusion compared to IV perfusion as a consequence of the sedimentation effects at the IO insertion sites [22]. Moreover, a study performed by Wong et al. [13] found that IO access and IV access were similar in terms of maximum concentration of adrenaline in animal models; nevertheless, they saw that IO access was inferior to IV access in terms of the time to maximum concentration [13]. These findings in animal studies might explain the results of some human studies that claim that IV administration of adrenaline is associated with better clinical outcomes.

### 4.2. Infusion Site Location

It is also important to acknowledge infusion site location differences and physiological characteristics during OHCA. Delguercio et al. [23] reported that during cardiopulmonary resuscitation, cardiac output approximately reaches 30% of its normal values. Consequently, blood flow is significantly decreased in OHCA patients, also when performing cardiopulmonary resuscitation. Since adrenaline is an α-receptor agonist that enhances coronary perfusion pressure during resuscitation [23,24], it might act faster when the infusion site is closer to the heart. This hypothesis was verified by a study conducted by Hoskins et al. [10] They showed that adrenaline delivered with a sternal IO access reached higher peak concentrations in a shorter period of time than a tibial IO pathway [10]. In a similar fashion, Beaumont and colleagues showed that adrenaline delivered with a humeral IO access reached higher maximum concentrations and led to higher odds of survival than with a tibial IO pathway [25]. In the majority of the OHCA cases, the IO accesses were established in the lower limbs, while the majority of the IV accesses were gained in the upper limbs, which is more proximal to the coronary circulation. This can potentially lead to bias when assessing the differences in medication administration between IO and IV access in OHCA [1,2,5,6,7,8,14,18,19]. It has also been suggested that chest compressions may increase the intrathoracic pressure and therefore impede venous return. This might decrease the effectiveness of drugs administered through IO routes established in the lower limbs [26,27].

## 5. Limitations

First of all, it is very difficult to study aspects of treatment in OHCA patients, since these are performed under very high stress levels, which can potentially hamper the quality of trials performed.

A total of nine studies were included in our systematic review. Of these studies, six were retrospective cohort studies, one prospective study, and two were sub-analyses of previous randomized trials, which represents an important drawback of the potential generalizability of the findings.

As pointed out in the systematic review performed by Granfeldt et al. [9] there seem to be better results in the group with IV access, but with very low certainty due to a variety of aspects, among them human and pharmacokinetics. As a consequence of the significant heterogeneity among the data of the studies, we chose not to do a meta-analysis.

## 6. Conclusions

In our systematic review, we have found a small number of studies comparing IV and IO administration of adrenaline during cardiac arrest. Due to significant heterogeneity, a meta-analysis was not performed, and based on current studies, no firm conclusions can be drawn about which route of adrenaline administration leads to better outcomes.

## Figures and Tables

**Table 1:** Characteristics of the included studies.

Author	Type of Study	N	Intervention	Primary Outcome	Secondary Outcome	Unadjusted Analysis First	Adjusted Analysis First	Unadjusted Analysis Second	Adjusted Analysis Second
Feinstein et al. [1]	Retrospective cohort study	N = 1800 IV 1525 IO 275	Primary route of vascular access: first patent access for drug administration IV vs. IO	Survival to hospital discharge	Sustained ROSC Survival to hospital admission	IO less likely to survive hospital discharge 14.9% vs. 22.8%, *p* = 0.003	No difference after adjusting for confounders in survival to discharge OR 95% CI 0.81, 0.55, 1.21, *p* = 0.31	IO less likely to achieve ROSC 43.6% vs. 55.5% *p* < 0.001 Or be hospitalized 38.5% vs. 50.0%, *p* < 0.001	IO access associated with lower likelihood of ROSC OR = 0.67 0.50, 0.88, *p* = 0.004 and survival to hospitalization OR 0.68 0.51, 0.91, *p* = 0.009
Clemency et al. [7]	Retrospective chart review of EMS records	N = 1310 IV 788 IO 552	First access type attempted First dose of parenteral epinephrine	ROSC at arrival ED	x	IO first approach non-inferior to IV first approach, ROSC 19.9% vs. 19.7%, *p* = 0.01 Epinephrine first IO vs. IV, ROSC 18.6% vs. 20.9% OR 0.86; 95% CI: 0.66–1.13	IO first approach non-inferior to IV first approach, ROSC 19.9% vs. 19.7%, *p* = 0.01 Epinephrine first IO vs. IV, ROSC 18.6% vs. 20.9% OR 0.86; 95% CI: 0.66–1.13	IO group superior 1st attempt success to IV group 81.6% vs. 94.8%, *p* < 0.01	
Kawano et al. [6]	Secondary analysis PRIMED study (Retrospective data analysis)	N = 13,155 IV 12,495 IO 660	Initial route of vascular access IV vs. IO	Favorable neurologic outcome on hospital discharge	ROSC Survival to hospital discharge	IO associated with decreased probability of favorable neurological outcome OR 0.22, 95% CI 0.12–0.42	Compared with IV, IO decreased probability of favorable neurological outcome OR 0.24; 95% CI 0.13–0.46	IO associated with decreased probability of ROSC OR 0.53, 95% CI 0.44–0.66 And survival OR 0.42 95% CI 0.28–0.63	Compared with IV, IO decreased probability of ROSC OR 0.60 95% CI 0.49–0.74 And survival 0.45 95% CI 0.29–0.69
Nguyen et al. [18]	Retrospective cohort study	N = 795 IV 453 IO 342	IV vs. IO access First access IV vs. IO (intention to treat)	ROSC		ROSC IV vs. IO 45.1% vs. 25.7%, *p* < 0.001 ROSC IV vs. IO first 42.4% vs. 26.6%	ROSC IV vs. IO 45.1% vs. 25.7%, *p* < 0.001 ROSC IV vs. IO first 42.4% vs. 26.6%		
Mody et al. [2]	Retrospective cohort study	N = 19,731 IV 16,663 IO 3068	Attempted IO vs. IV	Survival to hospital discharge	Rates of sustained ROSC Survival with favorable neurological outcome	IO vs. IV 4.6% vs. 5.7%, *p* = 0.01	IO no longer associated with decreased survival vs. IV OR 0.88 95% CI 0.72–1.09, *p* = 0.24	Favorable neurological status at discharge 2.8% vs. 4.2% Sustained ROSC IO vs. IV 17.9% vs. 23.5%	Favorable neurological status at discharge OR 0.87 95% CI 0.67–1.12, *p* = 0.29 Sustained ROSC IO vs. IV OR 0.80 95% CI 0.71–0.89, *p* < 0.001
Zhang et al. [5]	Retrospective observational analysis	N = 35,733 IV 27,758 IO 7975	First and only adrenaline route IV vs. IO	Survival to hospital discharge	ROSC Survival with good neurological outcome	IV vs. IO 5.8% vs. 3.1%, *p* < 0.05	OR of IV vs. IO 1.468 95% CI, 1.264–1.705	ROSC IV vs. IO 24.5% vs. 17.8%, *p* < 0.05 Survival with favorable neurological outcome IV vs. IO 4.3% vs. 1.8%, *p* < 0.05	ROSC IV vs. IO OR 1.367 95% CI, 1.276–1.464 Survival with favorable neurological outcome IV vs. IO OR 1.849 95% CI 1.526–2.240
Tan et al. [19]	Prospective parallel cluster-randomized study	N = 1016 IV only 478 IV + IO 529	IV route at scene (max 2 attempts) IV or IO at scene (max 2 attempts IV then IO)	Any ROSC	Insertion success rate Proportion of patients who received first dose of adrenaline Time to first dose of adrenaline Survival outcome	IV + IO vs. IV OR 0.99 95% CI 0.75–1.29	Post hoc per protocol analysis IV + IO 38.6% vs. 37.2%, *p* = 0.721	Success rate IV + IO vs. IV 76.6% vs. 61.1%, *p* = 0.001 Prehospital adrenaline IV + IO vs. IV 71.3% vs. 55.4%, *p* = 0.001 IV + IO faster Adrenaline 23 vs. 25 min, *p* = 0.001 Survival outcome IV + IO vs. IV 4% vs. 3.4%, *p* = 0.630	Post hoc per protocol analysis Success rate IV + IO vs. IV 100% vs. 61.1%, *p* < 0.001 Prehospital adrenaline IV + IO vs. IV 93.5% vs. 55.4%, *p* < 0.001 Survival outcome IV + IO vs. IV 4.9% vs. 8.4%, *p* = 0.054/3.3% vs. 4.0%, *p* = 0.713
Baert et al. [14]	Retrospective comparative multi-center study	N = 28,856 IV 27,280 IO 1576	IO vs. IV access	Survival at 30 days or hospital discharge	ROSC Survival at hospital admission Neurological outcome at day 30 or discharge	Survival day 30 1.9% vs. 3.8%, *p* < 0.001	Survival discharge or day 30 IO vs. IV 1.8% vs. 2.4%, *p* = 0.266	ROSC IO vs. IV 19.7% vs. 27.7%, *p* < 0.001 Survival at hospital admission IO vs. IV 14.8% vs. 23.4%, *p* < 0.001 Favorable neurological outcome 81.8% vs. 72.7%, *p* = 0.343	ROSC IO vs. IV 19.8% vs. 25.3%, *p* < 0.001 Favorable neurological outcome IO vs. IV 85.2% vs. 65.7%, *p* = 0.082
Nolan et al. [8]	Placebo controlled trial	N = 3631 IO 1116 IV 2515	IO vs. IV adrenaline vs. placebo	Survival at 30 days	ROSC at handover hospital Survival at discharge Favorable neurological outcome		aHR IV vs. IO within 1 day survival 1.02 95% CI 0.94–1.10 aHR IV vs. IO over 1 day survival 1.30 95% CI 0.98, 1.72		ROSC adrenaline vs. placebo IV aOR 4.07 95% CI 3.42–4.85 vs. IO aOR 3.98 95% CI 2.86–5.53, *p* = 0.90

Abbreviations: IV = intravenous; IO = intraosseous; ROSC = return of spontaneous circulation; EMS = emergency medical services; OR = odds ratio; aOR = adjusted odds ratio; HR = hazard ratio; aHR = adjusted hazard ratio; vs. = versus.

**Table 2:** Assessment of methodological quality using the Newcastle–Ottawa Scale (NOS) [16].

Criteria ^1^	S1	S2	S3	S4	C1	O1	O2	O3	T
Feinstein et al. [1]	*	-	*	-	*	*	*	*	6
Clemency et al. [7]	*	*	*	-	**	*	*	*	8
Kawano et al. [6]	*	*	*	-	**	*	*	*	8
Nguyen et al. [18]	*	*	*	*	**	*	*	*	9
Mody et al. [2]	*	*	*	*	**	*	*	*	9
Zhang et al. [5]	*	-	*	-	**	*	-	*	6
Tan et al. [19]	*	-	*	-	*	*	*	*	6
Baert et al. [14]	*	*	*	-	*	*	*	*	7
Nolan et al. [8]	*	*	*	-	*	*	*	*	7

^1^ Abbreviations: S1 = representiveness, S2 = selection, S3 = ascertainment, S4 = demonstration, C1 =comparability, O1 = outcome selection, O2 = outcome follow-up, O3 = adequacy. For criteria points S1–S4, O1–O3 it is possible to achieve 1 star, for criteria point C1, it is possible to achieve 2 stars. * The study suffices in this criteria point. ** The study suffices in these two criteria points.
